# The plantar proximal cortex of the third metatarsal bone shows raised longitudinal ridges at the suspensory ligament enthesis in normal equine isolated limbs – a radiographic, computed tomography, and MRI study

**DOI:** 10.3389/fvets.2023.1265116

**Published:** 2023-11-16

**Authors:** Michaël Dancot, Zoë Joostens, Fabrice Audigié, Valeria Busoni

**Affiliations:** ^1^Department of Medical Imaging, Faculty of Veterinary Medicine, Université de Liège, Liège, Belgium; ^2^Equitom - Equine Care Group, Lummen, Belgium; ^3^CIRALE, Ecole Nationale Vétérinaire d’Alfort, Goustranville, France

**Keywords:** suspensory ligament, enthesis, third metatarsal bone, radiography, computed tomography, horse

## Abstract

**Introduction:**

Knowledge of normal radiographic appearance is essential to avoid misinterpretation of radiographs. This study aimed to assess the computed tomographic (CT) appearance of the plantar surface of the proximal metatarsus and evaluate the influence of the radiographic angle on the trabecular/cortical interface of the proximal plantar metatarsal cortex on lateromedial and slightly oblique radiographs.

**Methods:**

Eight hindlimbs were collected from six horses with no known history of lameness and euthanized for reasons unrelated to the study. Limbs underwent computed tomographic (CT) and radiographic examination (dorsoplantar, lateromedial, and slightly oblique radiographic views obtained by angling the beam dorsally and plantarly from the plane used for the lateromedial projection). Standing magnetic resonance (MR) imaging and computed tomography (CT) were used to confirm normalcy. Images were compared side-by-side by two experienced readers.

**Results:**

Limbs were normal at MR imaging. Longitudinal linear ridges were present on the proximal plantar metatarsal surface in all limbs (1–2 sagittal ridges and 1 ridge located at the medial or lateral margin of the suspensory ligament). Longitudinal ridges were positioned facing an adipose-muscular bundle of the suspensory ligament on CT images and were visible as linearly increased opacities on dorsoplantar radiographs. The delineation of the trabecular/cortical interface of the proximal metatarsus changed with radiographic projection and was the sharpest on the plantaro 85° lateral to the dorsomedial oblique view.

**Conclusion:**

The proximal third metatarsal bone shows individual morphological variations, with longitudinal linear ridges that alter the bone homogeneity on dorsoplantar radiographs. An oblique plantaro 85° lateral to the dorsomedial view is suggested to better assess the presence of subcortical sclerosis when proximal suspensory enthesopathy is suspected.

## Introduction

Proximal suspensory desmopathy is a common cause of lameness in horses (1–3). This condition may be acute or progressive in nature, but regardless of the chronicity, it tends to manifest as decreased performance in both athletic and pleasure horses rather than in obvious lameness (4). Injury to the proximal suspensory ligament not only affects the ligament itself but also may affect the entire enthesis, including the proximal aspect of the third metatarsal bone (MtIII) (4–7). Considering the combination of soft-tissue and osseous damage that can be present, multiple imaging modalities are often necessary to fully characterize the lesions and plan the patient’s management (7). Radiography, ultrasonography, computed tomography (CT), and magnetic resonance imaging (MRI) are routinely used to assess morphological abnormalities (3, 8–10). Scintigraphy may be part of the work-up for lesion localization in horses with unclear clinical presentation and that show increased bone uptake at the enthesis (11).

In the field, prior to further advanced imaging investigation in selective cases, initial imaging assessment is performed via radiography and ultrasonography (12). A dorsoplantar radiographic projection is used to evaluate the area of the major component enthesis of the suspensory ligament that is located at the plantar proximal aspect of the MtIII (13), and a plantaro 75° lateral to dorsomedial oblique lateral oblique projection is considered preferable to a lateromedial image to complement the dorsoplantar view (16). The main radiographic abnormalities reported in the literature include increased radiopacity and/or linear lucencies of the proximal MtIII in the dorsoplantar view and subcortical sclerosis in the lateromedial view (3, 8, 12, 13). The findings with increased radiopacity have been described as consistent with “new bone formation at the origin of the suspensory ligament” (12) or “enthesophyte formation at the plantar aspect of the MtIII” (13, 15). No clear association has been found between linear lucencies on dorsoplantar radiographic views and bone resorption on MR images (12). However, the interpretation of radiographic changes in the proximal MtIII is considered challenging; given the high rate of false positive diagnosis on radiographs (12, 13), their correlation with the presence of proximal suspensory desmitis has been questioned (3, 7, 13). Moreover, on dorsoplantar radiographic views in normal limbs, increased lateral opacity of the proximal MtIII has been related to the largest thickness of the bone on its lateral aspect (12), and the presence of a ridge on its plantar aspect has been reported to be responsible for a central thin sclerotic line (17).

Therefore, this study aimed to explore the morphological variations of the plantar surface of the proximal MtIII that may mimic proximal suspensory enthesopathy on dorsoplantar radiographs and evaluate the influence of the radiographic angle on the appearance of its plantar cortex on lateromedial radiographs.

It was hypothesized that linear bands of increased opacity visible on the dorsoplantar view without associated endosteal sclerosis are related to the presence of longitudinal osseous ridges on the plantar surface of the normal MtIII and that the radiographic appearance of the trabecular/cortical bone interface of the plantar MtIII will change depending on the radiographic angle used, with the interface being sharpest at a beam angle tangential to the plantar MtIII surface.

## Materials and methods

### Limb selection

Hindlimbs were collected from warmblood horses euthanized at an equine hospital for reasons other than for the study. Horses were selected if there was no known history of lameness and were included if euthanized during the time period of the study. Limbs were amputated at the tibiotarsal joint and immediately refrigerated (4°C).

### Imaging examinations

Imaging examinations were performed within 5 days after euthanasia on the limbs brought to room temperature.

#### Computed tomography

A CT examination was performed using a 16 multi-slice helical scanner (Siemens, Somatom Sensation 16, Erlangen, Germany) with automatic acquisition parameters (tube voltage 120 kVp, automatically adapted tube current 110–180 mA, pitch 1, and total collimation width 1.2 mm). Raw data sets were reconstructed using a 512 × 512 matrix with a pixel spacing of 0.21 mm and a slice thickness of 0.6–1 mm. Two bone reconstruction windows were used (B70s sharp and U90u very sharp convolution kernels). The angle between a line tangential to the talar ridges drawn at their most dorsal extent and a line tangential to the trabecular/cortical bone interface of the proximal MtIII at approximately mid-height of the proximal suspensory enthesis was calculated based on transverse CT images using standard medical image viewing software (RadiAnt DICOM viewer, Medixant, 2009–2017, Poznan, Poland). This angle was used to obtain oblique radiographic projections with the beam tangential to the trabecular/cortical bone interface of the proximal MtIII.

#### Radiographic examination

**Figure 1:**
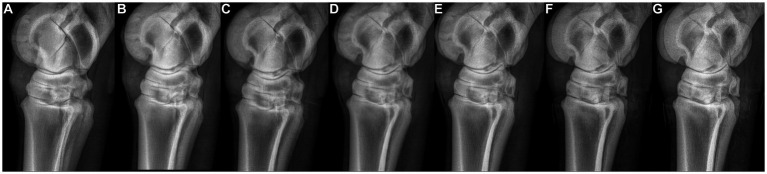
Radiographs of the proximal metatarsus obtained with different angles of incidence of the radiographic beam. From left to right: one projection angling the beam in a dorsolateral-plantaromedial direction at 5° and 2° from the reference plane of the lateromedial view [**(A,B)**, respectively], a strict latero-medial view **(C)**, and four projections angling the beam in a plantarolateral–dorsomedial direction at 2° **(D)**, 5° **(E)**, 7° **(F)**, and 10° **(G)**.

After CT, each limb was placed in lateral recumbency on the table of a small animal radiography unit (GE table, General Electric Medical Systems, Milwaukee, Wisconsin, USA and Agfa CR35 developer, Agfa Gevaert Healthcare, Mortsel, Antwerpen, Belgium). Fluoroscopy was used to position the limb in a radiographic view, perfectly lateromedial to the talar ridges (18). This lateromedial view was used as a starting position to angle the radiographic beam dorsally and plantarly to obtain further oblique radiographic views of the proximal MtIII. Tube angulation was verified on the control panel. The dorsoplantar view was obtained by placing the limbs on their plantar aspect on the same radiographic table. In total, eight views per limb were obtained as follows: lateromedial projection (0° – reference plane), two projections angling the beam in a dorsolateral–plantaromedial direction at 2° and 5° from the reference plane (dorso 88° lateral to plantaromedial oblique and dorso 85° lateral to plantaromedial oblique views), and four projections angling the beam in a plantarolateral–dorsomedial direction at 2°, 5°, 7°, and 10° (plantaro 88° lateral to dorsomedial oblique, plantaro 85° lateral to dorsomedial oblique, plantaro 83° lateral to dorsomedial oblique, and plantaro 80° lateral to dorsomedial oblique views) (Figure 1), and the dorsoplantar view.

#### Magnetic resonance imaging examination

Each limb was assessed using an MR imaging low-field system (0.27 T, Hallmarq Veterinary Imaging Ltd., Guildford, Surrey, United Kingdom) to confirm normalcy according to the previously described MR imaging appearance of the proximal suspensory ligament and the proximal MtIII (13). The protocol used was a combination of gradient echo sequences (T1 GRE, T2* oW) and spin echo (T2 FSE, STIR FSE) in the sagittal, dorsal, and transverse planes.

### Image analysis

DICOM images were viewed on a dedicated workstation using local software for archiving and reading medical images (Impax 6.6, AGFA, Belgium). MR and CT images and dorsoplantar radiographs were compared side-by-side in DICOM format by two experienced readers (an ECVDI Dip – VB and a last year ECVDI resident – MD). The position and number of the ridges visible on the CT images were noted and compared with the corresponding radiographic and MR images to localize the ridges on the dorsoplantar views and to determine their position relative to the suspensory ligament and its adipose-muscular bundles. To evaluate the sharpest delineation of the plantar cortex, lateromedial and oblique radiographs (56 views) were cropped by an independent operator to exclude the talar ridges and pasted in a random order, in JPEG format, on PowerPoint. Cropped JPG images were then compared with each other blindly and independently by the two readers (VB and MD) for the sharpness of the trabecular/cortical bone interface to identify images with the sharpest appearance, which were classified as sharp or very sharp. The cropped images with the sharpest appearance were selected, and the corresponding DICOM images that correlated with the radiographic view were used to obtain the projection angle.

## Results

### Horses and limbs

Eight hindlimbs (four left and four right) from six horses were included (Supplementary material 1). The horses included one mare, one stallion, and four geldings of various breeds (Belgian Warmblood, Selle Français, Westphalen, and Arabian crossbred) and disciplines (leisure, dressage, jumping, and unbroken). The median age of the horses was 4 years (age range 3–19 years). The weight of the horses ranged from 386 to 635 kg (median 520 kg). All limbs were normal at MR imaging and CT examination.

### Morphology of the proximal metatarsus at the suspensory ligament origin

**Figure 2:**
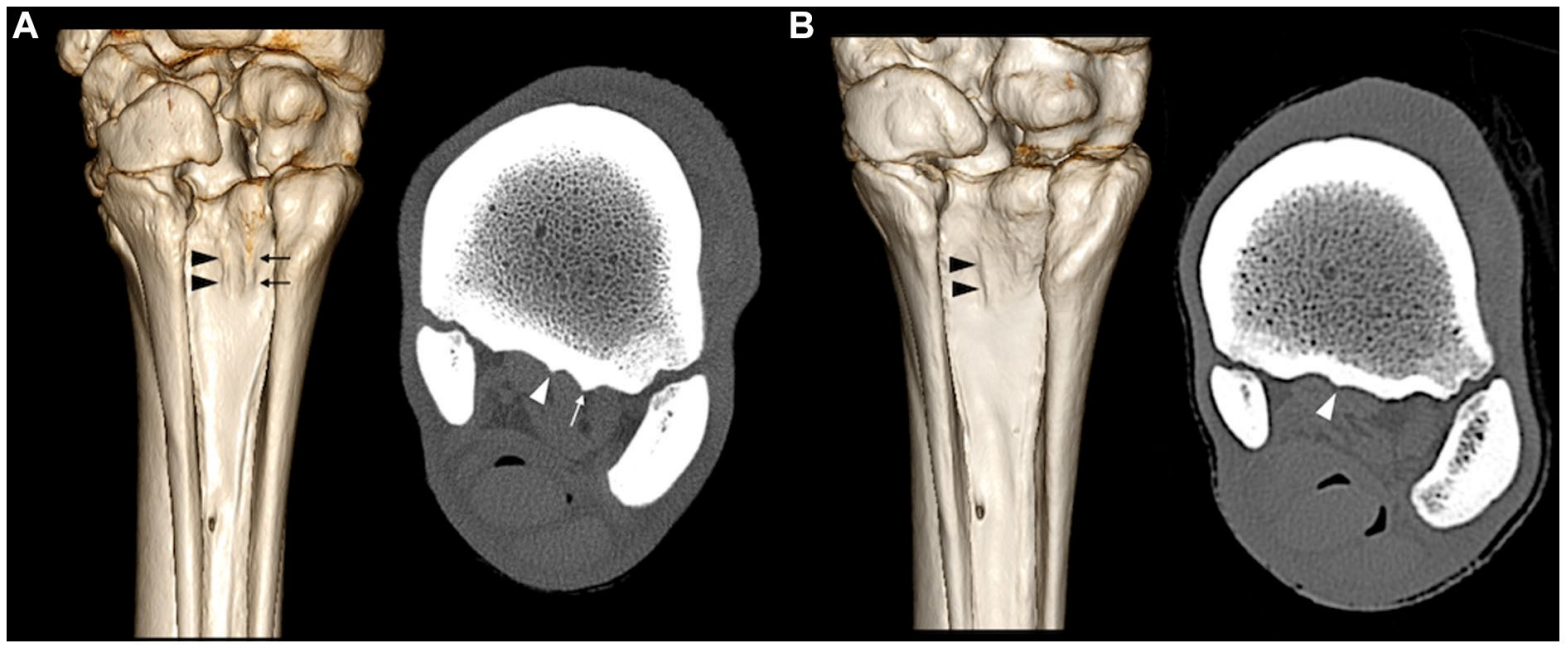
Volume rendering reconstructions and bone window computed tomography transverse reconstructions of two limbs **(A,B)** showing respective corresponding sagittal ridges (arrowheads) and lateral ridges (arrows) at the plantar surface of the MtIII.

**Figure 3:**
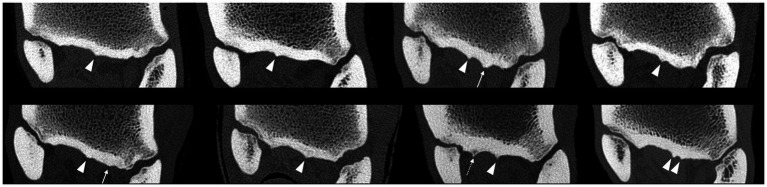
Bone window computed tomographic reconstructions in the transverse plane of the eight limbs showing the variations of the plantar surface of the MtIII among horses: all limbs show sagittal ridges (arrowheads), two limbs show one abaxial lateral ridge (arrows), and one limb shows one abaxial medial ridge (dashed arrow).

**Figure 4:**
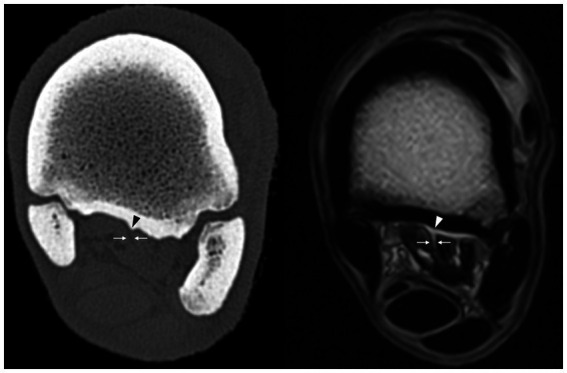
T1 GRE HR transverse magnetic resonance sequence image and bone window computed tomography transverse reconstruction of the same leg at the same level showing the non-ligamentous fatty component (arrows) of the suspensory ligament facing the ridge at the plantar surface of the third metatarsal bone (arrowheads).

**Figure 5:**
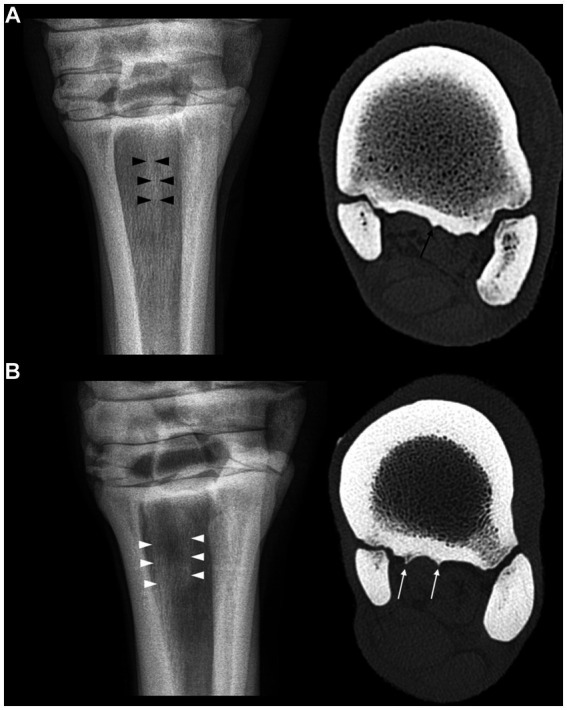
**(A)** Dorsoplantar radiograph of the proximal part of the third metatarsal bone showing a longitudinally oriented opacity band in its sagittal part (black arrowheads) related to the sagittal ridge in bone window computed tomography (black arrow). **(B)** Dorsoplantar radiograph of the proximal part of the third metatarsal bone showing two longitudinally oriented opacity bands in its sagittal part and medial part (white arrowheads) related to the sagittal and medial ridges in bone window computed tomography (white arrows).

On the CT images, the plantar surface of the MtIII showed more or less prominent longitudinal linear ridges on the proximal plantar metatarsal surface (Figure 2). All limbs showed one (seven limbs) or two (one limb) ridges in or very close to the mid-sagittal plane. Three limbs showed a second abaxially located ridge at the margin of the suspensory ligament (two lateral and one medial) (Figure 3). There were large individual variations in the height and position of the ridges. Two limbs showed faint ridges, but at least one ridge was always present. The plantar surface of the lateral aspect of the proximal MtIII was more irregular than the medial aspect in all limbs, independent from the height of the ridges. All ridges were positioned facing non-ligamentous tissue components of the suspensory ligament on CT and MR images (Figure 4) and were visible as linearly increased opacities on dorsoplantar radiographs (Figure 5). The sagittal ridges were positioned facing the partial sagittal cleft, dividing the suspensory ligament into lateral and medial lobes. CT and MR images showed large variations in size, shape, and distribution of the adipose-muscular bundles within the proximal suspensory ligament. Left and right hindlimbs from the same horse showed identical topographical mirror distributions of the plantar MtIII ridges and adipose-muscular bundles.

### Sharpness of the trabecular/cortical bone interface

**Figure 6:**
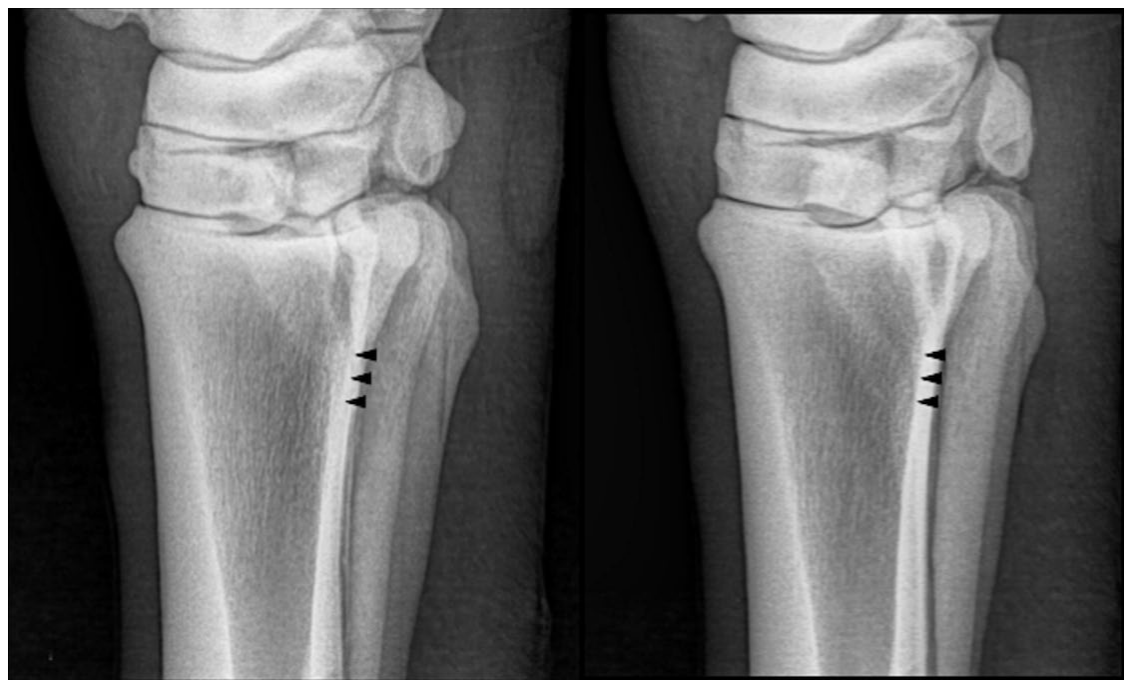
**(A)** Lateromedial and **(B)** plantaro 85° lateral to dorsomedial oblique proximal metatarsal radiographs of a normal horse. The trabecular/cortical bone interface is sharper in the plantaro 85° lateral to dorsomedial oblique projection.

On lateromedial and oblique radiographs, the sharpness of the trabecular/cortical bone interface of the plantar proximal MtIII changed depending on the projection angle. The sharpest interface was obtained in the plantaro 85° lateral to dorsomedial oblique view (Figure 6). The plantarolateral–dorsomedial oblique views were more often classified as sharp or very sharp (52 views out of the 64 plantarolateral–oblique views were classified as sharp or very sharp by both readers).

## Discussion

This study on a small number of isolated limbs shows that the proximal MtIII and the proximal hind suspensory ligament have a variable anatomy among horses. The plantar MtIII surface has one or two longitudinally oriented ridges, and the proximal suspensory ligament has adipose-muscular bundles of different sizes and shapes.

The suspensory ligament appearance agrees with previously described individual variations showing random areas of fat and muscle dissecting through the ligament (14, 15). Significant asymmetry between the left and right hindlimbs has been described (7), but left and right symmetry was not evaluated in the present study as both hindlimbs of only two horses were collected. However, in these two horses, the ridges and the adipose-muscular bundles had an identical topographical mirror distribution.

The hypothesis that linear bands of increased opacity seen on dorsoplantar radiographs may be related to the presence of longitudinally oriented ridges on the plantar metatarsal surface has been confirmed in all limbs. Every limb in the present study showed one or two longitudinal ridges at the plantar MtIII surface on CT, which appeared to correspond in location to the linear bands of increased opacity on dorsoplantar radiographs when compared side-by-side. This is in accordance with the description by Butler et al. of similar longitudinal bands of increased opacity on dorsoplantar radiographs as a normal radiographic feature (16). However, such longitudinal radiopaque bands have also been described as manifestations of linear sclerosis in response to an adaptive and/or pathological phenomenon at the enthesis of the suspensory ligament (12). In the present study, no subcortical sclerosis was observed in the selected limbs, and thus, the linear bands visible on radiographs seemed solely created by the plantar cortical ridges. Diffuse or multifocal increased opacities in the proximolateral aspect of the MtIII on dorsoplantar radiographs have also been reported as new bone formation or enthesophytes (13). In the present study, the longitudinal ridges observed on CT may correspond to a description of new bone formation or enthesophytes. However, because no abnormality in the suspensory ligament or in the subcortical trabecular bone was observed, neither on CT nor on MR images, a pathological nature was considered unlikely, and the longitudinal ridges were considered as anatomical variations.

The hypothesis that the sharpness of the trabecular/cortical bone interface changes with the angle of incidence of the radiographic beam has also been confirmed. The radiographic projection that better delineates the plantar MtIII cortex and its endosteal margin is the plantaro 85° lateral to dorsomedial oblique projection. This view not only significantly decreases the superimposition of the proximal part of the second and fourth metatarsal bones but is also obtained with a radiographic beam perfectly tangential to the trabecular/cortical bone interface. Such tangential orientation is essential to obtain sharp subcortical delineation because of the geometry of radiographic image formation. The importance of this concept has already been highlighted for the *facies flexoria* of the distal sesamoid bone, where dedicated radiographic projections tangential to the trabecular/compact bone interface have been suggested to improve the radiographic assessment of its proximal and distal aspects (19). At the proximal suspensory ligament enthesis, the angle of the radiographic projection is particularly crucial, as subcortical sclerosis is a radiographic feature suggestive of a radiographic diagnosis of proximal enthesopathy (12, 16). It can, therefore, be speculated that, if the radiographic projection is not taken into consideration, the number of false positive results may increase while evaluating routine lateromedial views of the proximal MtIII or hock region in clinical practice. It can also be speculated that, in the case of avulsion fracture or prominent bone production in enthesopathy, the plantaro 85° lateral to dorsomedial oblique projection will perform better than a routine lateromedial view in assessing the plantar surface of the MtIII.

During the routine assessment of the proximal suspensory ligament enthesis, ultrasound examination can identify the longitudinal ridges described in the present study, in particular, using a strict transverse approach perpendicular to the MtIII, with the fetlock in flexion and the foot on the ground (Busoni, personal communication). Because of their longitudinal orientation, these ridges will appear smooth on longitudinal ultrasound sections and are not misinterpreted as signs of enthesopathy in longitudinal sections.

In the limbs used for the present study, the lateral aspect of the plantar MtIII was more irregular than the medial aspect. Suspensory ligament lesions have been described as having a prevalent lateral location in the hindlimbs (3, 20). The more irregular lateral plantar surface of the MtIII may reflect increased biomechanical stresses on the lateral portion of the enthesis, as previously described (3, 21). However, a larger number of limbs than examined in the present study would be necessary to evaluate a potential correlation between stress/weight and the degree of irregularity in horses.

In both CT and MR images, the longitudinally oriented ridges were positioned facing non-ligamentous fatty components of the suspensory ligament. The sagittal ridges were positioned facing the cleft, separating the two lobes of the ligament more distally. In the human literature, fat (insertional angle or endotenon fat) is known to play an important role in stress dissipation, especially at entheses where the insertional angle significantly changes with movement (22). Such fat pads are often associated with the point of maximum convexity of the bony insertion (22). The plantar metatarsal ridges described in this study may thus reflect the normal surface of bony growths, dissipating stress away from adjacent collagenous parts of the proximal suspensory insertion. In the human literature, it is also described that to reduce wear and tear, the fibrocartilaginous enthesis develops a flat surface to minimize the risk of damaging the surrounding tendinous tissue (23). This fibrocartilaginous nature has been recently proposed while describing the histological features of the enthesis of the suspensory ligament in equine hindlimbs (24). If the suspensory ligament enthesis is considered fibrocartilaginous, the longitudinal ridges may therefore be the result of an adaption process of the bone component between the two flat areas dedicated to the fibrocartilaginous entheses of the ligamentous parts. However, this hypothesis must be histologically investigated further by comparing the bone-suspensory ligament attachment in areas facing the tendinous part of the ligament with attachment in areas facing the fatty portions of the ligament.

The number of isolated limbs used in the present study was small, and the only inclusion criteria for the horses were not having a previous known history of hindlimb lameness and having undergone euthanasia for medical reasons unrelated to the present study. Therefore, the limbs were harvested from a relatively inhomogeneous small sample, and the included hindlimbs likely do not represent the full spectrum of individual variations.

Neither macroscopic dissection nor histology was made on the limbs, and standing MR imaging and CT were used to confirm normalcy. Therefore, minor abnormalities, not detectable with these imaging modalities, may have been present in the entheses of the hindlimbs selected. However, it is unlikely that standing (post-mortem) MR imaging and CT had missed clinically significant lesions, and if undetected microscopical lesions were present, these were likely similar to subclinical lesions present in non-lame horses.

Finally, the legs were refrigerated for a few days before the MR imaging assessment. It has been shown that changes are minimal after refrigeration and may create variations in the signal-to-noise ratio, leading to minimal changes in STIR and FSE sequences (25). However, overall MR image quality is considered unchanged in limbs refrigerated for less than 14 days and after a freezing-thawing cycle (26). For the present study, it was therefore considered that preservation conditions were unlikely to affect the results.

In conclusion, this study demonstrates that longitudinally oriented bone ridges are present on the plantar surface of the proximal MtIII at the suspensory ligament enthesis in the hindlimbs of warmblood horses. It also illustrates that horses show individual morphological variations not only of the adipose-muscular bundles in the hind suspensory ligament but also of these longitudinal bony ridges on the plantar MtIII surface. Because these ridges can alter the homogeneity of bone opacity and mimic sclerosis on the dorsoplantar radiographic view, a slightly (5°) plantarolateral-to-dorsomedial oblique view, tangential to the trabecular/cortical bone interface instead of a strict lateromedial view perfectly superposing the talar ridges, is suggested to better assess the presence of subcortical sclerosis as a radiographic sign of proximal suspensory ligament enthesopathy.

## Data availability statement

The original contributions presented in the study are included in the article/Supplementary material, further inquiries can be directed to the corresponding author/s.

## Ethics statement

Ethical approval was not required for this study in accordance with the local legislation and institutional requirements because the research was made on isolated limbs from euthanized animals for reason unrelated to the study. Written informed consent was not obtained from the owners for the participation of their animals in this study because it was undertaken on isolated limbs from horses whose owner gave consent for necropsy.

## Author contributions

MD: Visualization, Writing – original draft, Writing – review & editing, Conceptualization, Data curation, Investigation. ZJ: Supervision, Writing – original draft, Writing – review & editing, Conceptualization, Data curation, Investigation, Validation. FA: Supervision, Writing – review & editing, Conceptualization & validation. VB: Funding acquisition, Supervision, Validation, Writing – original draft, Writing – review & editing, Conceptualization, Investigation, Methodology, Resources, Data curation.
